# Passive standing as an adjunct rehabilitation intervention after stroke: a randomized controlled trial

**DOI:** 10.1186/s40945-015-0002-0

**Published:** 2015-07-08

**Authors:** Francesco Ferrarello, Gabriella Deluca, Assunta Pizzi, Carlo Baldini, Francesca Iori, Niccolò Marchionni, Mauro Di Bari

**Affiliations:** 1 Functional Rehabilitation Unit, Azienda USL 4, Via Cavour 118/120, 59100 Prato, Italy; 2 Fondazione Don Carlo Gnocchi ONLUS-IRCCS, Florence, Italy; 3 Research Unit of Medicine of Aging, Department of Experimental and Clinical Medicine, University of Florence, and Azienda Ospedaliero-Universitaria Careggi, Florence, Italy

**Keywords:** Stroke, Supported standing, Randomized controlled trial

## Abstract

**Background:**

Early physical rehabilitation enhances functional recovery in stroke survivors. Supported standing is a common adjunctive therapeutic practice in subjects with several central nervous diseases who are unable to stand actively. Data on the effect of supported positioning on standing frames in individuals with recent stroke are scarce and contradictory.

**Objectives:**

To verify if the addition of supported standing practice (SSP), delivered by means of a standing frame in two durations, to conventional physical therapy (CPT), may improve motor function, autonomy, and mobility in individuals with disability due to recent stroke.

**Methods:**

After baseline assessment, 75 participants with severe disability due to stroke, all receiving CPT, were randomly assigned to adjunctive 20 or 40 min of SSP, or CPT only (control). Motor function, autonomy, and mobility were assessed before and after training, and three months later.

**Results:**

All participants assessed received the planned dose of intervention. No adverse events of SSP were detected. Most outcome measures improved from baseline through the end of treatment and in the follow-up in all groups; the extent of change was comparable across the three randomization groups.

**Conclusions:**

In this randomized trial, SSP was unable to provide any sizeable adjunctive benefit, above and beyond CPT, in subjects with recent stroke.

## Background

Stroke is a major cause of disability and death worldwide [1], with only a small proportion of survivors achieving full functional recovery after acute event. Indeed, at 40 % of survivors have difficulties in performing basic self-care tasks after 6 months [2], and more than 30 % report participation restrictions as long as 4 years after stroke [3]. Systematic reviews show that organized multidisciplinary care and rehabilitation are effective in the early phase of a stroke, improving survival and independence, and reducing length of hospital stay and need for institutionalization [4].

Physical rehabilitation enhances substantially the functional recovery in stroke survivors, and improvements are greater with earlier beginning and higher intensity of rehabilitation [5]. Early mobilization lessens the likelihood of acute phase complications (e.g., pneumonia, pressure sores, deep vein thrombosis) and usually begins when clinical conditions are stable [6]. Beyond sitting, the standing position brings additional benefits such as prevention of hip and knee flexors contractures, circulatory training, autonomic nervous system stimulation, and sensory activation [7]. Moreover, recovery of the ability to stand up and sustain load on the affected limb is crucial to gait training and recovery of upper limb functionality [8, 9]. Supported standing on tilt table or standing frame is an adjunctive therapeutic practice commonly adopted in subjects with several central nervous diseases who are unable to stand actively. Its aim is to improve antigravity muscles strength and head and trunk postural control, maintain standing ability, and prepare for gait training [10].

Data on the effect of supported positioning on standing frames in patients with recent stroke are scarce and contradictory. In a randomized controlled trial (RCT), 14 sessions of supported standing, added with flexible duration and schedules to conventional rehabilitation within nine weeks after stroke onset, did not significantly improve functional status [11]. However, potential weaknesses should be recognized in this study: the intervention was administered late in the course of the disease, its intensity was low, its duration was left to the therapists’ discretion [11], and the first post-treatment assessment often did not coincide with the end of the intervention [10]. In another pilot RCT, an intervention combining passive standing on a variety of stabilizers and task training, achieved some improvement in balance [12]. Thus, we deemed as necessary a study conducted with more stringent selection criteria and a rigorously controlled intensity of the intervention, in order to ascertain the merit of passive standing in post-stroke rehabilitation.

We conducted the present RCT to verify whether the addition to early conventional physical therapy (CPT) of supported standing practice (SSP) delivered by means of a standing frame in two durations, improves motor function, autonomy, and mobility in individuals with disability due to recent stroke.

## Methods

The reporting of this study conforms to the CONSORT (Consolidated Standards of Reporting Trials) Statement for non-pharmacologic trials [13]. The study was approved by the IRCCS Fondazione Don Gnocchi ethics committee.

### Subjects

Persons with recent stroke, admitted for early rehabilitation to the inpatient Neurology Unit of the IRCCS Fondazione Don Gnocchi in Florence, Italy, from April 2011 to February 2012, were screened for enrollment. Inclusion criteria were: (1) first ever ischemic or hemorrhagic stroke, (2) age ≥18 years, (3) admission within 4 weeks from stroke onset, (4) severe functional limitation in walking (score 0 or 1 in the Functional Ambulation Classification, FAC) [14], (5) tolerance to the standing frame of at least 10 min (assessed two days after admission), (6) stable clinical conditions, (7) adequate participation and cognitive capacity, and (8) ability to provide informed consent.

Exclusion criteria were: (1) clinical contraindications to prolonged upright position (e.g. postural hypotension), (2) previous stroke, (3) severe limitations of the range of motion, particularly the lack of hip and knee extension, and ankle dorsiflexion, (4) the presence of recent fractures of the pelvis or lower limb (if full weight- bearing was not allowed) and (5) any other co-morbidity or disability that would preclude participation in the training program. Eligible subjects signed an informed consent conforming to the Helsinki Declaration, which contained detailed information on study design and data management.

### Study design and procedures

After inclusion and baseline assessment, participants were stratified according to the FAC score (0 and 1). They were then randomly assigned to either adjunctive 20 or 40 min of SSP (experimental interventions), or standard (see below) CPT only (control). The random sequence was generated by an investigator not involved in participants’ assessment (MDB) using a web-based application (http://www.randomizer.org). Allocation assignment was kept concealed using serially numbered, opaque, sealed envelopes.

Baseline and follow-up evaluations were performed by an independent physical therapist (GD), blinded to group allocation and otherwise uninvolved in the study. Treatments were administered by physical therapists five days a week for three weeks, in an inpatient setting. Assessments were done at enrollment (T0), at the end of the 3-week treatment (T1), and three months later (T2).

A formal sample size calculation was not performed because of lack of adequate preliminary data on the expected effect size. In particular, the present RCT differed from previous studies in the outcome measures chosen, in the overall duration of the treatment period and in the double dosing (20 and 40 min/day) of the intervention.

### Experimental treatment

Participants randomized to the 40-min daily SSP treatment received 2 20-min sessions (morning and afternoon). The standing frame STANDY (Ormesa®, www.ormesa.com) was utilized for the experiment. Equipped with heel rests, knee pads and back rest (both adjustable in width, depth, and height) it allows a flexible and safe static regimen.

Patients’ positioning on the standing and monitoring were conducted by the treating physical therapists. Exercise duration was timed and inconveniences or discomforts were recorded. The standing session was not performed, or could be interrupted, if participants showed intolerance or hypotension.

### Conventional physical therapy treatment

All participants underwent individual CPT sessions, which included 60 min of neuromuscular and musculoskeletal interventions, and practice of functional activities [15]. Moreover, 20 min of passive cycling (upper- and/or lower- limbs), hydrokinetic and occupational therapy were administered, as well as cognitive and speech therapy when needed.

### Data

Demographic and main clinical characteristics (stroke type, days from event, and affected side) were recorded. Stroke severity was assessed with the National Institute of Health Stroke Scale (NIHSS). The clinical history of participants was investigated for any contraindications to upright position. Goniometric measurement of the range of movement of the lower limbs was performed to exclude limitations that might affect the ability to stand. Moreover, the ability to control the trunk in an upright position (sitting and standing) was recorded. Tolerance to the standing frame was assessed by measuring blood pressure, heart and respiratory rate first in the supine and seated positions, and then after 5 min of standing. Signs and symptoms of hypotension were monitored. As a safety measure, blood pressure and heart rate were recorded also during the interventions.

#### Primary outcome measures

The Fugl-Meyer Assessment of Motor Recovery after Stroke - motor domain (FM) scale, the Functional Independence Measure (FIM), and the FAC were chosen as primary outcome measures.

The FM, used in both clinical and research settings, is one of the most widely used quantitative measures of motor impairment, with excellent intra- and inter- rater reliability and construct validity, particularly of the motor domain, as shown by numerous studies [16]. The motor domain includes items measuring movement, coordination, and reflex action about upper- and lower- limb; motor score ranges from 0 (hemiplegia) to 100 points (normal motor performance), divided into 66 points for the upper extremity and 34 points for the lower extremity. The FIM measures the level of disability and indicates how much assistance is required to perform activities of daily living [17]. The FAC categorizes subjects according to basic motor skills necessary for functional ambulation [14].

#### Secondary outcome measures

The Modified Ashworth scale (MAS), the Timed-Up-and-Go test (TUG) at three months (T2), the drop in systolic blood pressure from supine to standing position, and the ability to control the trunk while sitting or standing were taken as secondary outcome measures.

The MAS [18] evaluates spasticity in patients with lesions of the central nervous system; knee flexors and extensors, ankle dorsal and plantar flexors, and hip adductors of the affected side were assessed. The TUG is a widely used mobility test, which requires that a person rises from a chair, walks three meters, turns around, walks back to the chair, and sits down again on the chair, while being timed [19].

Moreover, the Trunk Control Test (TCT) [20], which examines four simple aspects of trunk movement, was added to the protocol after the study began, and was therefore administered in a subsample.

### Statistical analysis

Data were analyzed by an independent investigator, blind to group allocation. Sample characteristics were analyzed by descriptive statistics. Differences between groups in baseline demographic and pre-training characteristics were examined using one-way ANOVA for continuous and the chi-square test for ordinal and categorical variables respectively, taking into account trends as appropriate. To perform the between-group comparisons, a two-way ANOVA for repeated measures with group x time interaction was used. The Kruskal-Wallis test was utilized to highlight the between-group differences in the TUG test three months after the end of treatment.

Analyses were performed according to the intention- to- treat (ITT) and the per- protocol (PP) principles [21]. For ITT analysis missing data were dealt with by using the last- observation- carried- forward method [21]. The IBM SPSS Statistics for Windows, (version 20.0; IBM Corp, Armonk, NY) was utilized for calculations. The significance level was set at a p value of <0.05.

## Results

### Characteristics of the sample

**Fig. 1:**
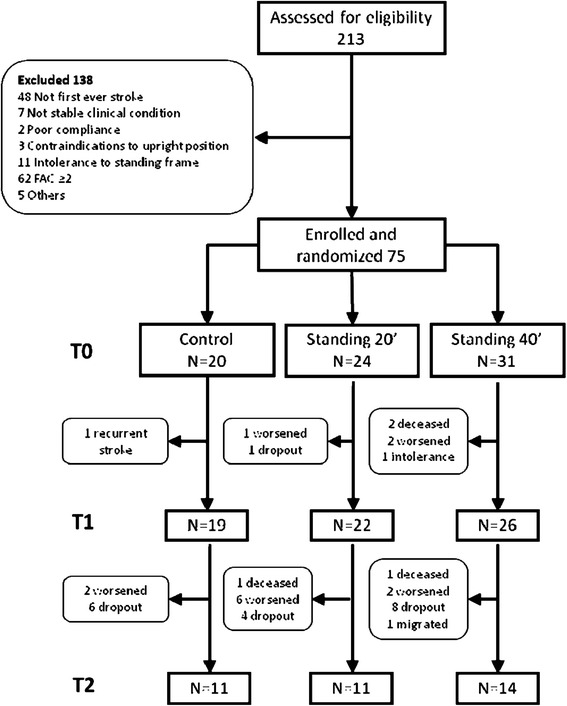
Study flow chart. Abbreviations: FAC = Functional Ambulation Categories; T0 = baseline; T1 = end of treatment; T2 = follow-up (3 months)

Of 213 individuals with recent stroke screened, 138 did not fulfill the enrolment criteria and were therefore excluded (Fig. 1), leaving a final sample of 75 participants (37 females), who were randomly assigned to 40 min SSP (*n* = 31), 20 min SSP (*n* = 24), or control (*n* = 20). Age ranged from 18 to 97 years (mean 74.0); days from event ranged from 5 to 22 days (mean 12.3). Cases of hemorrhagic etiology, as well as of right-sided hemispheric lesion, were 27 (36 %); at baseline 36 (49 %) participants were not able to manage trunk control while standing, and 56 (72 %) could not ambulate or could ambulate only on parallel bars (FAC category 0). Baseline participants’ characteristics were comparable across the three randomization groups (Table 1). At the end of the 3-week treatment period (T1) and three months later (T2), 67 and 36 participants were assessed, respectively (Fig. 1). All subjects assessed received the planned dose of intervention and had complete data. No adverse events occurred; one participant withdrew because of intolerance to the standing frame.

**Table 1:** Baseline characteristics of the study groups

	Control	20’ SSP	40’ SSP	P
	(*n* = 20)	(*n* = 24)	(*n* = 31)	
Variable				
Age	75.15 (±3.64)	76.65 (±1.78)	72 (±2.62)	0.430
Days from event	13.5 (±0.92)	12.13 (±0.86)	11.87 (±0.69)	0.562
Gender F/M	8/12	14/10	16/15	0.476
Type of stroke I/E	10/10	17/7	21/10	0.305
Lesion side DX/SX	6/14	10/14	11/20	0.722
FAC 0/1	14/6	20/4	20/11	0.297
Trunk control upright No/Yes	7/13	12/11	17/14	0.353
NIHSS	7.70 (±1.02)	8.71 (±0.97)	8.68 (±0.82)	0.716
FM	10.55 (±1.11)	8.89 (±1.19)	8.33 (±0.91)	0.600
FIM	53.65 (±3.83)	51.17 (±2.85)	49.45 (±2.89)	0.708
SBP supine	127.75 (±3.44)	129.17 (±3.06)	123.28 (±4.51)	0.752
DBP supine	72.25 (±2)	72.08 (±1.7)	76.61 (±2.09)	0.673
SBP standing	123.75 (±3.44)	124.17 (±3.06)	123.28 (±4.51)	0.916
DBP standing	72.50 (±2.16)	69.79 (±1.69)	74.31 (±1.99)	0.674
MAS	3.5 (±0.43)	4.26 (±0.5)	4.14 (±0.48)	0.538
TCT^a^	55.50 (±8.96)	45.79 (±8.52)	46.19 (±7.94)	0.681

Data are mean (standard error) and proportions for continuous- and ordinal- or categorical- variables respectively

*20’ SSP* 20 min Supported Standing Practice, *40’ SSP* 40 min Supported Standing Practice, *DBP* Diastolic Blood pressure, *FAC* Functional Ambulation Categories, *FIM* Functional Independence Measure, *FM* Fugl-Meyer Assessment of Motor Recovery after Stroke - motor domain, *NIHSS* National Institute of Health Stroke Scale, *MAS* Modified Ashworth Scale, *SBP* Systolic Blood Pressure, *TCT* Trunk Control Test

^a^Sample at T0: Control = 12, 20’ SSP = 14, 40’ SSP = 16

**Fig. 2:**
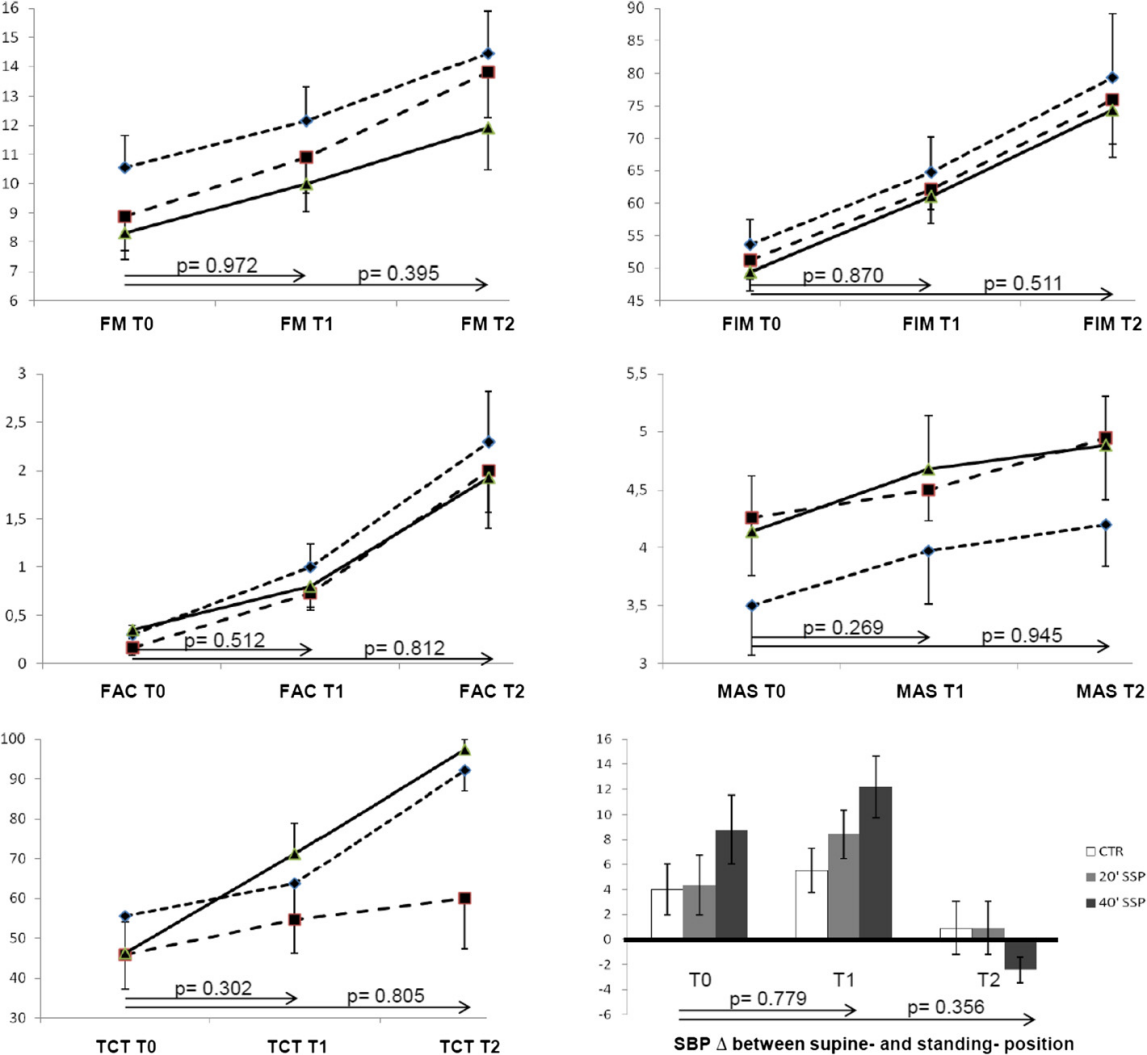
Mean outcome measures score as a function of time in the three groups. Error bars represent the standard error. The TCT was administered in a subsample. Figure legend. CTR = Control;  20’SSP = 20/min Supported Standing Practice;  40’SSP = 40/min Supported Standing Practice. Abbreviations. As in Table 2

**Fig. 3:**
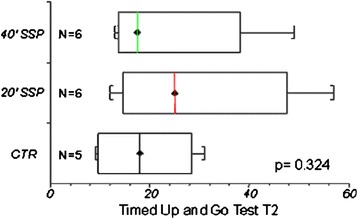
T2 Timed Up and Go Test, between-groups comparison

The results of the ITT and PP analysis were comparable. The data shown in tables and figures refer to the PP analysis. As a whole, all participants significantly improved their condition. In between-group comparisons, the study groups obtained similar scoring in final assessment in all outcome measures, both at the end of treatment and three months later (Table 2, Figs. 2 and 3).

**Table 2:** Effects of experimental and conventional interventions

	Control Group			20’ SSP Group			40’ SSP Group		
Variable	T0 (n=20)	T1 (n=19)	T2 (n=11)	T0 (n=24)	T1 (n=22)	T2 (n=11)	T0 (n=31)	T1 (n=26)	T2 (n=14)
FM	10.55 (±1.11)	12.16 (±1.14)	14.45 (±1.46)	8.89 (±1.19)	10.9 (±1.23)	13.82 (±1.55)	8.33 (±0.91)	10 (±0.97)	11.93 (±1.46)
FIM	53.65 (±3.83)	64.79 (±5.44)	79.3 (±9.85)	51.17 (±2.85)	62. 15 (±3.18)	75.92 (±6.84)	49.45 (±2.89)	61.08 (±4.2)	74.43 (±7.4)
FAC	0.3 (±0.1)	1 (±0.24)	2.3 (±0.52)	0.17 (±0.08)	0.73 (±0.18)	2 (±0.43)	0.35 (±0.09)	0.8 (±0.22)	1.93 (±0.53)
MAS	3.5 (±0.43)	3.97 (±0.46)	4.2 (±0.36)	4.26 (±0.5)	4.5 (±0.27)	4.95 (±0.54)	4.14 (±0.48)	4.68 (±0.46)	4.89 (±0.42)
TCT*	55.5 (±8.96)	63.83 (±9.15)	92.2 (±5.2)	45.79 (±8.52)	54.57 (±8.33)	60 (±12.64)	46.19 (7.94)	71.17 (±7.7)	97.4 (±2.6)
SBP Diff	4 (±2.04)	5.53 (±1.79)	0.91 (±2.11)	4.35 (±2.4)	8.42 (±1.95)	0.91 (±2.11)	8.79 (±2.75)	12.2 (±2.47)	-2. 43 (±1.03)

Data are mean (standard error)

*20’SSP* 20/min Supported Standing Practice, *40’SSP* 40/min Supported Standing Practice, *FAC* Functional Ambulation Categories, *FIM* Functional Independence Measure, *FM* Fugl-Meyer Assessment of Motor Recovery after Stroke - motor domain, *MAS* Modified Ashworth Scale, *SBP Diff* difference in Systolic Blood Pressure between the supine and standing position, *T0* baseline, *T1* end of treatment, *T2* follow-up (3 months), *TCT* Trunk Control Test

^a^The TCT was administered in a subsample (Control/20’ SS/40’ SSP: T0 = 12/14/16, T1 = 12/13/12, T2 = 5/7/5)

## Discussion

Supported standing programs are routinely used in many settings, different clinical conditions and age groups [22]. Being passively supported in the upright position by adaptive equipment is a common intervention for individuals who have inadequate postural control or lower limb strength [10]. The stroke rehabilitation community showed interest in the topic, in fact in a systematic review exploring the issue in adults with upper motor neuron injury, slightly more than half of the participants in the studies included had stroke [10]. Evidence related to the effectiveness of SSP in neurorehabilitation is controversial and few firm conclusions can be drawn from systematic reviews, limited to some positive effects reported in the presence of stabilized symptoms [10, 22, 23].

This study shows that the addition of SSP for 20 or 40 min to CPT, administered in an early phase, does not improve motor function, autonomy, and mobility in individuals with disability due to a stroke. Although outcome measures improved significantly from baseline through the end of treatment and in the follow-up, the extent of change was unrelated to the assignment group. At the same time, the SSP had no adverse effects.

These findings are consistent with those of the largest previous study on this issue [11], which had been considered as inconclusive because of methodological limitations, such as late application and poor standardization of the intervention [10, 11].

In our study, we tried to overcome some of the weaknesses that limited previous experiments [10–12], by narrowing the time gap to enrollment, standardizing and increasing the dose administered, and conducting the first post-treatment assessment soon at the end of the intervention. We administered the adjunctive SSP within 22 days from event and with a precise delivered dose, which might reflect clinical practice for adopted session duration, frequency, and assistive device [10].

As the comparisons with other studies are difficult, because baseline participants’ disability level or type of treatment administered were not similar [12, 24, 25], it was not possible to estimate the sample size in our RCT and, therefore, it is possible that our study is under-powered. However, the absence of any trend towards improvement and of a dose-effect relationship (40 min vs. 20 min SSP) in any of our outcome measures discourages the design of further RCTs with larger sample size.

## Conclusions

In conclusion, this RCT failed to show any functional benefit of SSP in the rehabilitation of recent stroke. Nevertheless , enough support exists for the use of a standing device as part of a comprehensive 24-h postural management and activity program for individuals chronically ill with severely limited mobility [23]. We cannot exclude that, as shown in other neurological conditions such as multiple sclerosis [26] or cerebral palsy [23], SSP might provide some benefit in the stabilized phase of the disease, to limit the impact of long-term complications on overall well-being and quality of life [27, 28]. However, large, high quality studies are required to definitively verify these potential, late positive effects, which have so far limited support from scientific evidence.
